# Immunomodulatory effects of a probiotic combination treatment to improve the survival of Pacific oyster (*Crassostrea gigas)* larvae against infection by *Vibrio coralliilyticus*

**DOI:** 10.3389/fimmu.2024.1380089

**Published:** 2024-04-08

**Authors:** Jennifer Hesser, Ryan S. Mueller, Chris Langdon, Carla B. Schubiger

**Affiliations:** ^1^ Department of Biomedical Sciences, Carlson College of Veterinary Medicine, Oregon State University, Corvallis, OR, United States; ^2^ Department of Microbiology, College of Science, Oregon State University, Corvallis, OR, United States; ^3^ Coastal Oregon Marine Experiment Station and Department of Fisheries, Wildlife, and Conservation Sciences, College of Agricultural Sciences, Oregon State University, Corvallis, OR, United States

**Keywords:** Pacific oyster larvae, probiotics, immune response, *Vibrio coralliilyticus*, aquaculture

## Abstract

**Introduction:**

The culture of Pacific oysters (*Crassostrea gigas*) is of significant socio-economic importance in the U.S. Pacific Northwest and other temperate regions worldwide, with disease outbreaks acting as significant bottlenecks to the successful production of healthy seed larvae. Therefore, the current study aims to describe the mechanisms of a probiotic combination in improving the survival of *C. gigas* larvae. Specifically, we investigate changes in *C. gigas* larval gene expression in response to *V. coralliilyticus* infection with or without a pre-treatment of a novel probiotic combination.

**Methods:**

Treatment groups consisted of replicates of Pacific oyster larvae exposed to a) a combination of four probiotic bacteria at a total concentration of 3.0 x 10^5^ CFU/mL at 18 hours post-fertilization (hpf), b) pathogenic *V. coralliilyticus* RE22 at a concentration of 6.0 x 10^3^ CFU/mL at 48 hpf, and c) the probiotic combination at 18 hpf and *V. coralliilyticus* RE22 at 48 hpf. RNA was extracted from washed larvae after 72 hpf, and transcriptome sequencing was used to identify significant differentially expressed genes (DEGs) within each treatment.

**Results:**

Larvae challenged with *V. coralliilyticus* showed enhanced expression of genes responsible for inhibiting immune signaling (i.e., *TNFAIP3*, *PSMD10*) and inducing apoptosis (i.e., *CDIP53*). However, when pre-treated with the probiotic combination, these genes were no longer differentially expressed relative to untreated control larvae. Additionally, pre-treatment with the probiotic combination increased expression of immune signaling proteins and immune effectors (i.e., *IL-17*, *MyD88*). Apparent immunomodulation in response to probiotic treatment corresponds to an increase in the survival of *C. gigas* larvae infected with *V. coralliilyticus* by up to 82%.

**Discussion:**

These results indicate that infection with *V. coralliilyticus* can suppress the larval immune response while also prompting cell death. Furthermore, the results suggest that the probiotic combination treatment negates the deleterious effects of *V. coralliilyticus* on larval gene expression while stimulating the expression of genes involved in infection defense mechanisms.

## 1 Introduction

Today’s aquaculture production satisfies half of the global seafood demand, and this proportion is increasing as harvests of wild fish and shellfish populations reach maximum sustainable yields (1). In 2021, commercial oyster production in the U.S. totalled 23.9 million pounds, valued at 222.5 million USD (2). Hatcheries that rear oyster larvae and supply oyster farmers with seed periodically face disease outbreaks caused by pathogenic bacteria. Infections by pathogenic *Vibrio* spp. (vibriosis) are primarily responsible for mass mortalities, which can cause significant bottlenecks in oyster larval production and subsequent economic losses (3).

Several *Vibrio* species have been reported to cause disease outbreaks in bivalve aquaculture, including *V. aestuarianus, V. coralliilyticus, V. splendidus, V. tapetis, V. tasmaniensis*, and *V. tubiashii* (4). *Vibrio coralliilyticus* is one of the most prominent pathogens in this industry, being linked to numerous mortality events and showing virulence towards a range of host species, including the Pacific oyster (*Crassostrea gigas*), Eastern oyster (*Crassostrea virginica*), Kumamoto oyster (*Crassostrea sikamea*), and Geoduck clams (*Panope abrupta*) (3, 5–8). Symptoms of vibriosis in larvae are often characterized by a rapid decline in larval motility, detachment of the velum, and soft tissue necrosis (9, 10). Although clinical symptoms have been observed, the oyster larvae’s immune and inflammatory response following pathogenic infection are not well understood.

As invertebrates, oysters deploy an innate immune response to protect themselves from pathogenic bacteria, viruses, and parasites (11). Pattern recognition receptors (PRRs) bind to pathogen-associated molecular patterns (PAMPs), stimulating downstream signalling pathways. Several immune signalling pathways may be employed to produce immune effector molecules, cytokines, and antimicrobial peptides (12). Unfortunately, even with a defence response, oyster larvae are still found to be highly susceptible to pathogens (13).

Methods for preventing bacterial infections in oyster larvae typically include maintaining good water quality (i.e., frequent water changes, ozonisation, ultra-violet radiation treatment) and, occasionally, adding antibiotics (14). Unfortunately, water treatments are usually not effective enough to reduce the impacts of pathogenic bacteria, and antibiotic treatments are often prohibited due to environmental concerns (15, 16). Thus, researching alternative disease control methods, such as probiotics, is a priority. In recent years, several putative probiotics have been investigated to protect oyster larvae from pathogenic bacteria (17–21). In addition, a new probiotic combination treatment introduced by Madison et al. (22) improved the relative percent survival of *C. gigas* larvae by 65.95% when challenged with *V. coralliilyticus.* Additionally, exposure to the probiotic combination significantly improved size and settlement rates of *C. gigas* larvae (22). The authors suggested a form of immune priming as one potential mechanism responsible for these changes. This study builds on this preliminary work and aims to characterize the defence response of *C. gigas* larvae exposed to *V. coralliilyticus*, either with or without pre-exposure to a combination of these novel probiotics.

## 2 Material and methods

### 2.1 Bacterial cultures

Three of the four probiotic strains used in this study (B11, DM14, and D16) were previously isolated and evaluated as a single combination treatment (22). The DM14 and D16 isolates were identified as different *Pseudoalteromonas* spp., and B11 as an *Epibacterium* sp (22). The fourth strain (ASW1) was later isolated from larvae cultured in autoclaved seawater using methods outlined previously (22). Genetic identification of ASW1 was completed using 16s rRNA gene sequencing and the NCBI’s BLAST suite (23). The 16S rRNA sequence was submitted to GenBank (Accession OQ595186). ASW1, in combination with B11, DM14, and D16, was evaluated for efficiency in improving the survival of *C. gigas* larvae challenged with *Vibrio coralliilyticus* using well-plate assays as described by Madison et al. (22). To obtain isolates for larval assays, cells from glycerol stocks stored at -80°C were streaked on plates with seawater-based Luria-Bertani agar (LBSw; 10 g of tryptone, 5 g of yeast extract, and 15 g of agar, per liter of 10 µm filtered seawater) and incubated at 25°C for 48 hours. Colonies were grown in 5 mL of LBSw broth (10 g of tryptone, 5 g of yeast extract per liter of filtered seawater) at 25°C and agitated at 40 RPM on a roller drum (New Brunswick TC-7; New Brunswick Scientific, Enfield, CT, USA) for 24 hours. Cultures were then washed twice with autoclaved seawater by centrifuging at 3900 x g for 5 min and resuspended in autoclaved seawater. The optical density was measured at 600 nm (OD_600_) (Beckman DU 530, Beckman Coulter, Brea, CA, USA). Assuming that an OD_600_ measurement of 1.0 equalled approximately 8.0 x 10^8^ CFU/mL, bacterial cultures were appropriately diluted to obtain target concentrations of 6.0 x 10^3^ CFU/mL for *V. coralliilyticus* and 7.5 x 10^4^ CFU/mL for each probiotic strain of the combination treatment used in larval challenge experiments.

### 2.2 Larval rearing

Adult *C. gigas* were provided by the Molluscan Broodstock Program (MBP) at Oregon State University’s Hatfield Marine Science Centre. Oysters from genetically unrelated families were chosen for spawning. Oysters were held for two to four weeks in a conditioning system at 20°C to induce gametogenesis and continuously fed on a 50/50 (by cell concentration) mixed algal diet of *Isochrysis galbana* and *Chaetoceros neogracile* (total concentration of 10,000 cells/mL). When sexually mature, oysters were strip-spawned, and resulting gametes were fertilized following aseptic techniques (24). Larvae were hatched in sterile 250 mL Erlenmeyer flasks containing autoclaved seawater at 25°C, 32 ± 2 ppt salinity, and a pH of 8.2 ± 0.1. The seawater used to hatch larvae was pumped from the Yaquina Bay, Newport, passed through sand filters followed by 10-µm bag filters, and aerated with carbon-dioxide-stripped air overnight to adjust the pH before autoclaving. After the seawater was autoclaved, it was agitated and aerated on a shaker table (Benchmark, Sayreville, NJ, USA) at 80 RPM for 16 to 24 hours before adding fertilized eggs.

### 2.3 Effects of probiotic additions against *Vibrio coralliilyticus* at different times during early larval development

Our previous survival assays evaluated the effects of various probiotic combinations and concentrations in protecting developing *C. gigas* larvae against *Vibrio coralliilyticus* (22 and unpublished data*)*. In this present study, the effects of the timing of probiotic additions on larval survival were evaluated by dosing larvae with the probiotics at different times post-fertilization of the eggs.

Following egg fertilization, embryos were transferred from a concentrated egg suspension in a single sterile 1 L beaker to sterile 250 mL flasks containing autoclaved seawater at a final concentration of 50 embryos per mL. This concentration allowed for the rearing of sufficient numbers of healthy normal larvae for the assays while considering handling constraints. The flasks were agitated and aerated on a shaker table at 50 RPM throughout the culturing the of larvae. Treatment groups were designated by the timing of probiotic addition. Probiotics were provided to larvae at 2 (2 hr PB Only), 6 (6 hr PB Only), 12 (12 hr PB Only), 18 (18 hr PB Only), or 24 hpf (24 hr PB Only). The four probiotics (ASW1, B11, DM14, D16) were added to larval cultures as a combination treatment at a concentration of 7.5 x 10^4^ CFU/mL per probiotic strain (22). Each of these five probiotic treatment groups was cultured in triplicate flasks. Twenty-four hours post-fertilization (hpf), D-larvae from each flask were poured onto a sterile 40 µm sieve, rinsed with autoclaved seawater, and resuspended in probiotic-free autoclaved seawater, except for treatment groups receiving probiotics at 24 hpf. This 24 hpf treatment represented a “standard” method used in our earlier studies (22), whereby the probiotics were added to the larval cultures at 24 hpf, but the larvae were not resuspended in probiotic-free autoclaved seawater before the addition of *V. coralliilyticus*. Therefore, by adding the probiotic combination at time points before 24 hpf, we investigated if the probiotic additions could influence the larvae at different points early in larval development. The transfer of larvae into probiotic-free autoclaved seawater also limits potential interactions between the probiotics and pathogen within the culture environment and focuses on interactions within the gut of the host where infection begins.

**Figure 1:**
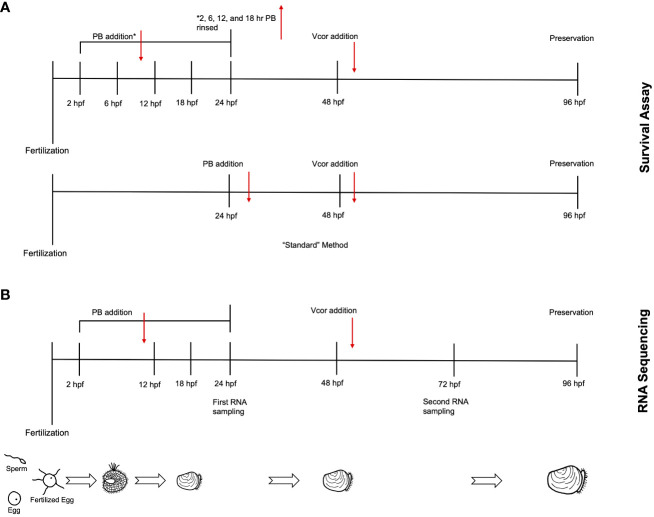
Experimental timeline for larval assays. **(A)** In the survival assay, probiotic treatment groups were designated by the timing of probiotic addition. Following fertilization, probiotics were added at either 2 (2 hr PB Only), 6 (6 hr PB Only), 12 (12 hr PB Only), or 18 hpf (18 hr PB Only). At 48 hpf, *Vibrio coralliilyticus* was added to probiotic-treated larvae (“2 hr PB + Vcor”, “6 hr PB + Vcor”, “12 hr PB + Vcor”, “18 hr PB + Vcor”, and “24 hr PB + Vcor”). A positive control of larvae receiving only *V. coralliilyticus* was inoculated at 48 hpf. An additional treatment group (24 hr PB Only) received probiotics at 24 hpf, which remained within the larvae culture until 96 hpf. (see methods). **(B)** For RNA sequencing, larvae were sampled from a similar yet separate experimental setup from that shown in **(A)** – see details in section 2.4. Larvae used for RNA extractions were collected from the “Larvae Only” control, the “Vcor Only” control, the “2 hr PB Only”, “12 hr PB Only”, “18 hr PB Only”, and the “2 hr PB + Vcor”, “12 hr PB + Vcor”, and “18 hr PB + Vcor” treatment groups at 72 hpf.

After transferring larvae from the 2, 6, 12, and 18 hpf treatments to probiotic-free autoclaved sea water, the larvae were resuspended at a concentration of 35 larvae/mL. Then one mL of larvae suspension was transferred to each well of a 24-well plate (**Figure 1A**). Larval concentrations were reduced in the well plates to better compensate for the addition of bacteria and subsequent influence on abiotic factors (22). Once transferred into well plates, the 24 hpf probiotic treatment groups received the probiotic addition.

At 48 hpf, probiotic-treated larvae were challenged with 6.0 x 10^3^ CFU/mL of *V. coralliilyticus* strain RE22 and incubated for 48 hours (these treatment groups were designated “2 hr PB + Vcor”, “6 hr PB + Vcor”, “12 hr PB + Vcor”, “18 hr PB + Vcor”, and “24 h PB + Vcor”). A positive control consisted of larvae infected with *V. coralliilyticus* alone without exposure to probiotics (Vcor Only). In addition, a negative control of larvae that received neither the probiotic treatment nor the pathogen was included (Larvae Only). Each treatment group consisted of six replicate wells (**Table 1**). At 96 hpf, larvae were preserved with 10 µL of 10% phosphate-buffered formalin at a pH of 8. Well-plates were observed under a 40X objective inverted light microscope. Larvae were counted as either live or dead, with larvae classified as dead, having less than 90% of their tissue remaining in their shells (22).

**Table 1:** Treatment groups and the timing of their bacterial additions throughout the larval assay.

Treatment Group	Probiotics added at time (hpf)	*V. coralliilyticus* added at time (hpf)
Larvae Only	–	–
Vcor Only	–	48
2 hr PB Only	2	–
6 hr PB Only	6	–
12 hr PB Only	12	–
18 hr PB Only	18	–
*24 hr PB Only	24	–
2 hr PB + Vcor	2	48
6 hr PB + Vcor	6	48
12 hr PB + Vcor	12	48
18 hr PB + Vcor	18	48
*24 hr PB + Vcor	24	48

*These larvae were exposed to probiotics from 24 hpf to 96 hpf, while larvae in all other probiotic treatment groups were rinsed at 24 hpf regardless of the duration of PB incubation^11^ii Larvae Only, V. coralliilyticus Only (Vcor Only), 2 hr Probiotics Only (2 hr PB Only), 6 hr Probiotics Only (6 hr PB Only), 12 hr Probiotics Only (12 hr PB Only), 18 hr Probiotics Only (18 hr PB Only), 24 hr Probiotics Only (24 hr PB Only), 2 hr Probiotics Only + V. coralliilyticus (2 hr PB + Vcor), 6 hr Probiotics Only + V. coralliilyticus (6 hr PB + Vcor), 12 hr Probiotics Only + V. coralliilyticus (12 hr PB + Vcor), 18 hr Probiotics Only + V. coralliilyticus (18 hr PB + Vcor), 24 hr Probiotics Only + V. coralliilyticus (24 hr PB + Vcor). ii. The addition of probiotics at 24 hpf was utilized as a standard method for evaluating probiotic effectiveness (22). The “-” symbol indicates the lack of bacterial addition for the indicated treatment.

### 2.4 Effects of bacterial exposure on larval gene expression

One liter flasks containing 500 mL autoclaved seawater were stocked with 1 x 10^4^ embryos. This concentration was chosen to ensure that adequate amounts of live larvae were collected for RNA analysis (4). The flasks were agitated and aerated on a shaker table at 50 RPM throughout the culturing of the larvae. The experiment was conducted with four replicates per treatment group. The probiotic combination of ASW1, B11, DM14, and D16 was added to the embryos at 18 hpf (18 hr PB Only) at a total concentration of 3.0 x 10^5^ CFU/mL. Unlike in the survival assay (**Figure 1A**), the larvae cultures were not rinsed and added to probiotic-free autoclaved seawater to minimize experimental effects on transcriptional changes and potential contamination and to increase the timing accuracy of bacterial exposures across all treatment groups and replicates. *Vibrio coralliilyticus* was then added to probiotic-treated larvae at 48 hpf (18 hr PB + Vcor). The larvae of the positive control received no probiotics, but *V. coralliilyticus* was added at 48 hpf (Vcor Only) (**Figure 1B**). A “no exogenous bacteria” negative control of larvae receiving no probiotic additions or *V. coralliilyticus* (Larvae Only) was maintained throughout this experiment.

All treatment and control groups were sampled at 72 hpf to ensure sufficient numbers of live larvae for RNA analysis after exposure to *V. coralliilyticus*. Five thousand larvae per treatment group were rinsed onto a sterile 40-µm sieve with autoclaved seawater and then rinsed with 2 mL of RNAlater (Thermo Scientific, Waltham, MA, USA). The larvae were then transferred to a microcentrifuge tube containing 1.5 mL RNAlater. In addition, samples of up to 100 larvae were collected from each of the four replicates into 10 mL shell vials (Thermo Scientific, Waltham, MA, USA) and preserved with 10% phosphate-buffered formalin (pH of 8) to evaluate survival, as described above.

### 2.5 RNA extraction, cDNA library preparation, and sequencing

Total RNA was extracted from each sample and DNase treated using the Zymo Direct-Zol RNA Microprep kit (Zymo Research, Irvine, CA, USA) with the addition of Trizol reagent (Thermo Scientific, Waltham, MA, USA). Total RNA extracted from each sample was quantified using the Qubit Broad Range RNA kit (Thermo Scientific, Waltham, MA, USA). The quality of total RNA was assessed using Agilent 2100 BioAnalyzer (Agilent, Santa Clara, CA, USA) with the Agilent RNA 6000 Nano kit (Agilent, Santa Clara, CA, USA). Typically, one of the four replicates within each treatment group had poor quality or quantity of total RNA; therefore, the three replicates from each treatment group with the highest quality and quantity of RNA were identified and prepared for sequencing.

The first step in sequence library preparation was the isolation of mRNA from the total RNA using the NEBNext Poly(A) Magnetic Isolation Module (New England BioLabs, Ipswich, MA, USA). Subsequently, complementary DNA (cDNA) libraries were constructed using the NEBNext Ultra II RNA Library Prep kit for Illumina (New England BioLabs, Ipswich, MA, USA), per the manufacturer’s instructions. Complementary DNA library quantification, normalization, and sequencing were then completed by Oregon State University’s Center for Quantitative Life Sciences (Corvallis, OR). Libraries were sequenced as 100-bp single-end reads with the NextSeq 2000. The P2 flow cell was utilized, allowing for 400 million reads to be sequenced per run. Four sequencing runs were completed, resulting in a sequencing depth of 32 million to 44 million reads per sample (**Supplementary Table 1**).

### 2.6 Assembly, annotation, and differential expression analysis

Raw sequencing reads were analysed for quality using FastQC 0.11.9 (25). Any identified poor-quality bases (quality scores lower than 20) and adapter sequences were removed using *fastp* (26). Processed reads were aligned to a *C. gigas* reference genome (version 1.0, Genbank GCA_902806645.1) via Hisat2 2.1.0 (27). Transcriptome assembly was performed using Stringtie (28) using default parameters. The overall alignment rate to the *C. gigas* reference genome using HISAT2 ranged from 45-80% for transcriptomes (**Supplementary Table 1**). Differential gene expression analysis was performed by comparing transcript counts between the treatment groups (“18 hr PB Only”, “18 hr PB + Vcor”, and “Vcor Only”) and the control (Larvae Only) using DESeq2, which was used also to normalize libraries to control for differences in sequence depth and remove outliers (29). Transcripts displaying a log fold change of ≥ 2 or ≤ −2 and a Benjamini-Hochberg adjusted *p*-value <.05 were considered significantly differentially expressed. A principal component analysis (PCA) was performed to observe the level of variation between treatment groups due to their level of differentially expressed genes. For significance testing of the PCA, a PERMANOVA was completed using the adonis2 function (30). Differentially expressed genes were annotated using KEGG’s GhostKOALA (KEGG Orthology and Links Annotation) program (31). Defence-related genes were annotated using Uniprot (32).

### 2.7 Statistical analyses

The “Larvae Only” negative control was used to normalize larval mortalities that were unrelated to experimental treatments. Relative percent survival (RPS) was calculated as RPS = [1-(percent mortality of treatment group/percent mortality of untreated control group)] x 100. Relative percent survival values were arcsine square root transformed before analysis. Statistical analyses were conducted using R statistical software (Version 4.0.3, R Project for Statistical Computing). Normality was assessed using the Shapiro-Wilk test and Q-Q plots. The homogeneity of variance was assessed using Levene’s test.

Nonparametric methods were used if violations of normality and variance assumptions were observed. For multiple comparisons of treatment groups, the Kruskal-Wallis one-way ANOVA was conducted. When significant differences (*P* <.05) were found, Dunn’s test with the Benjamini-Hochberg correction was used for pairwise comparisons. Comparisons between the two treatment groups were conducted using the Mann-Whitney U test.

## 3 Results

### 3.1 Identification of ASW1 as a probiotic bacterium

In evaluating ASW1 as a potential addition to the probiotic combination treatment, ASW1 did not result in any mortalities when added to larvae at 24 hpf (**Supplementary Table 2**). Larvae that received ASW1 at 24 hpf and *V. coralliilyticus* at 48 hpf had an average relative percent survival of 76.9 ± 38.9%, which was 56.1% higher than that of larvae exposed to *V. coralliilyticus* alone (**Supplementary Table 2**). A BLASTN suite search identified ASW1 as an *Alteromonas* sp. based on 16S rRNA sequencing. Specifically, *Alteromonas oceani* was identified as the best match with a 99% query coverage and 98.71% percent identity.

### 3.2 Effects of the probiotic combination on the survival of *V. coralliilyticus*-infected *C. gigas* larvae

**Figure 2:**
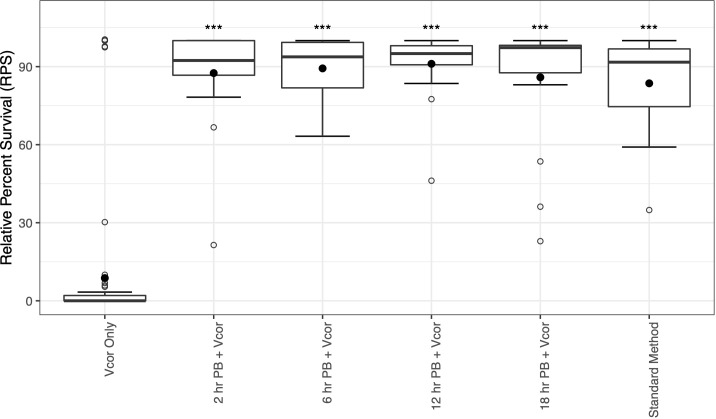
Relative percent survival of 4-day-old D-larvae of *C. gigas* challenged with *Vibrio coralliilyticus* strain RE22 with or without probiotic supplementation. “Vcor Only” was the positive control for survival and did not receive any probiotics. A negative control (Larvae Only) did not receive any probiotics or the pathogen. Filled circles represent the average relative percent survival of replicate wells (n=18). The boxes indicate the upper and lower quartiles and the bar represents the median or middle quartile. The ends of the whiskers represent the most extreme values within the 1.5x interquartile range (IQR), and the empty circles indicate outliers. *** indicates statistical differences from “Vcor Only” at *P* ≤.001.

Additions of the probiotic combination treatment at 2, 6, 12, 18, and 24 hpf were evaluated for the ability to reduce larval mortalities due to *V. coralliilyticus*. The negative control of larvae (Larvae Only) that received no bacterial additions and was used to calculate the relative percent survival yielded a survival rate of 99.7%. The positive control receiving only *V. coralliilyticus* (Vcor Only) resulted in an average relative percent survival of 8.7 ± 26.7% (**Figure 2**). When probiotics were added before pathogen exposure at 2, 6, 12, 18, or 24 hpf (PB + Vcor), the average relative percent survival significantly increased to 87.5 ± 18.9%, 89.3 ± 11.4%, 91.1 ± 12.9%, 85.9 ± 23.3%, and 83.6 ± 18.1%, respectively (*P* <.05) (**Figure 2**; **Supplementary Table 3**). There was no significant difference in survival among the different timings of probiotic additions (**Supplementary Table 3**); therefore, the 18-hour probiotic addition time was chosen as the focus for the RNA sequencing results to limit the possibility for changes in bacterial composition prior to the larvae being transferred into culture 24-well plates at 24 hpf.

### 3.3 Differential expression of genes compared with the larvae-only control

**Figure 3:**
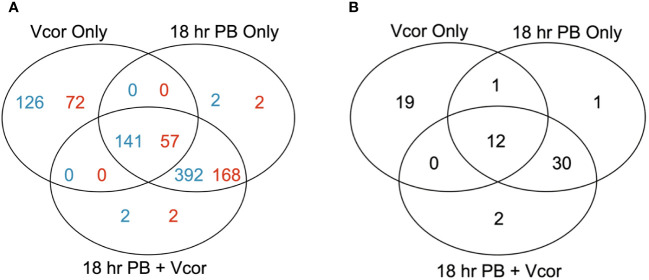
Numbers of differentially expressed genes in infection challenge and probiotic supplementation experiments. Venn diagrams represent the number of significant differentially expressed genes (DEGs) in each treatment group (“18 hr PB”, “18 hr PB + Vcor”, and “Vcor Only”) when compared to the “Larvae only” control. **(A)** The total numbers of all DEGs from each treatment, with the number of annotated genes listed in blue and the number of uncharacterized genes listed in red. **(B)** The total numbers of defence-related DEGs for each treatment compared to “Larvae only” controls.

Larvae infected with *V. coralliilyticus* without the probiotic supplementation (Vcor Only; positive control for disease) had 267 differentially expressed genes (DEGs) compared to larvae that received no bacterial additions (Larvae Only; negative control) (**Figure 3A**). One hundred and twenty-nine DEGs were annotated. When the probiotics were added to larvae at 18 hpf without any pathogen (18 hr PB Only), 535 DEGs were identified, including 227 annotated DEGs (**Figure 3A**). The same number, though not an identical set, of DEGs were identified when *V. coralliilyticus* was added in addition to the probiotics (18 hr PB + Vcor) compared to the negative control (Larvae Only) (**Figure 3A**) (**Supplementary Table 4**). The “Vcor Only” treatment group had 125 total unique DEGs, 72 being annotated. Comparatively, the “18 hr PB Only” and “18 hr PB + Vcor” each had two unique annotated DEGs and two unique uncharacterized DEGs. Between all three treatment groups, a total of 141 DEGs were shared, 57 of them being annotated genes.

When only defence-related DEGs were assessed, 32 DEGs were identified in the “Vcor Only” control compared to the “Larvae Only” control (**Figure 3B**). In comparison, there were 44 defence-related DEGs in both the “18 hr PB Only” and “18 hr PB + Vcor” treatment groups, 30 of which were identical (shared between both of the probiotic supplementation treatments). Conversely, the “Vcor Only” treatment group had 19 defence-related DEGs that were uniquely expressed in that treatment (**Figure 3B**). Twelve defence-related DEGs were shared between all three treatment groups (**Supplementary Table 4**).

### 3.4 Effects of the probiotic combination and *V. coralliilyticus* on the differential expression of defence-related genes

Defence-related genes that were identified as significantly differentially expressed were organized into four categories: pattern recognition receptors, immune signalling, immune effectors, and other immune or inflammatory genes. These groups describe various components of the larva’s innate immune system that recognize foreign material and respond accordingly. For instance, recognition of bacteria is accomplished by pattern recognition receptors, which include both extra- and intracellular protein receptors. Subsequently, the immune signalling category includes genes coding for proteins that contribute to relaying a signal throughout various immune pathways involving toll-like signalling, nuclear factor- kappa B, mitogen-activated protein kinase, complement cascade, and others. Genes identified as immune effectors respond to upstream signalling and contribute to the defence response. Finally, the genes grouped into the “other” category may contribute to the defence response through other routes, such as apoptosis or phagocytosis.

#### 3.4.1 Pattern recognition receptors

**Figure 4:**
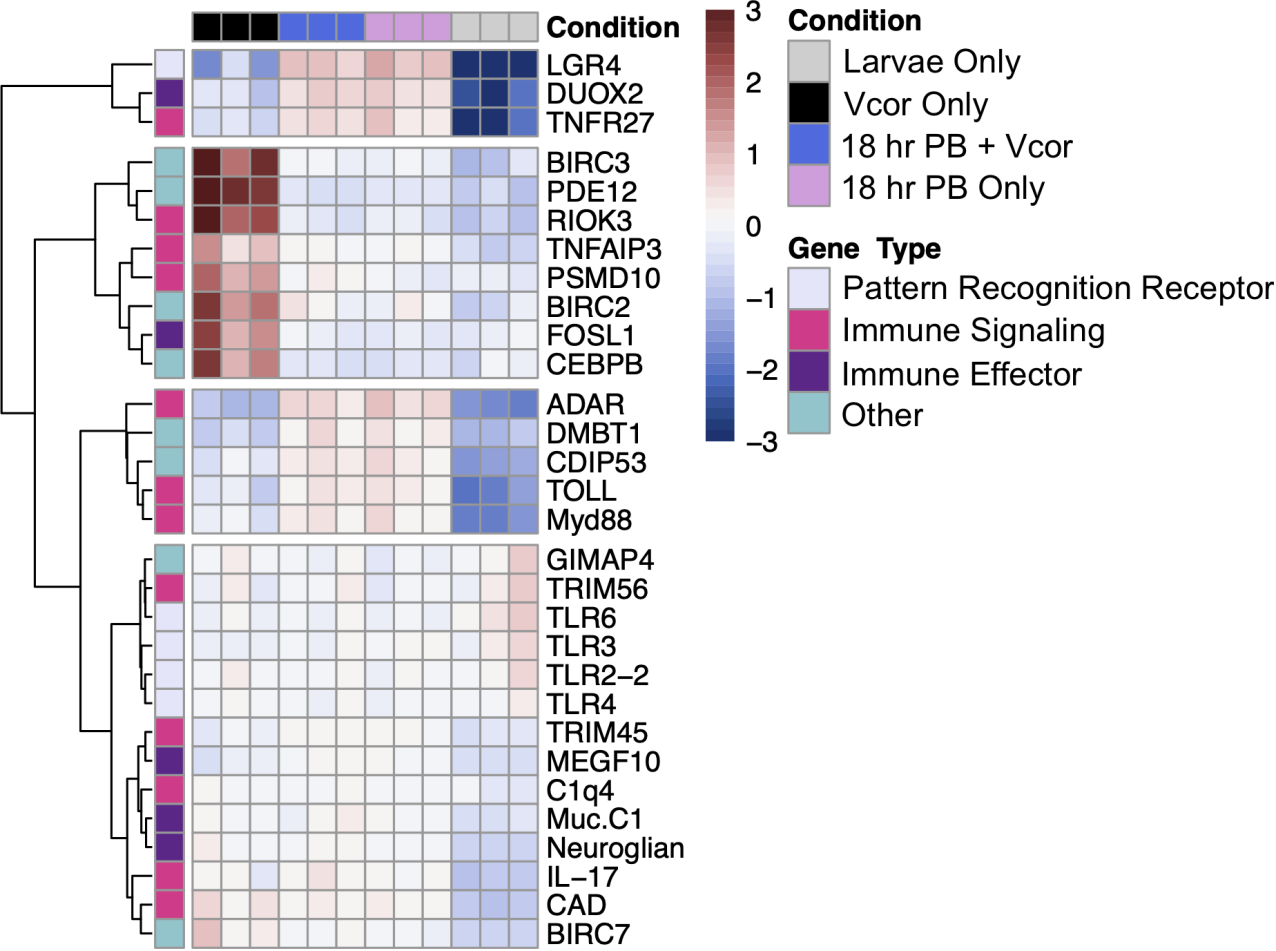
Differential gene expression of defence-related DEGs between the treatment groups (“Vcor Only”, “18 hr PB + Vcor”, and “18 hr PB Only”) and the negative control (Larvae Only). The treatment type is indicated at the top of the heatmap, with black signifying the “Vcor Only” infection control, blue the “18 hr PB + Vcor” treatment, pink the “18 hr PB Only” treatment, and grey representing the “Larvae Only” negative control. All samples were taken at 72 hpf, 24 hours after the addition of *V. coralliilyticus* to the “Vcor Only” and “18 h PB + Vcor” treatment groups. Heatmap and hierarchical clustering of selected genes is based on normalized read counts. Each column represents a single sample, with each row indicating the expression level of each gene. The colours represent the individual read count data are normalized to its average expression across all samples, with blue indicating lower than the genes’ average (decreased gene expression) and red indicating higher than the average (increased gene expression) (Gene names with corresponding abbreviations are found in **Supplementary Table 6**).

Two-day-old larvae previously exposed to bacterial additions (probiotic, pathogen, or both) had an increased expression of a gene that encodes the leucine-rich repeat-containing G-protein coupled receptor 4 (*LGR4*), a protein that behaves as a negative regulator of toll-like receptors (**Supplementary Table 5**). In contrast, the toll-like receptor 6 gene (*TLR6*) was found to be differentially decreased in expression in response to all three bacterial exposure treatments (“Vcor Only”, “18 hr PB + Vcor”, and “18 hrs + PB Only”) (**Figure 4**).

However, larvae that received the addition of only the probiotic combination (18 hr PB Only) showed a decreased and increased expression of the genes encoding the toll-like receptors 2 and 4 (*TLR2-2*, *TLR4*), respectively (**Figure 4**). In comparison, larvae exposed to *V. coralliilyticus* alone (Vcor Only) instead showed a decrease in the expression of the toll-like receptor 3 gene (*TLR3*) (**Supplementary Table 5**). Lastly, larvae exposed to the probiotics in addition to *V. coralliilyticus* (18 hr PB + Vcor) showed a similar gene expression pattern to the “18 hr PB Only” treatment group, presenting a decreased expression of the *TLR2-2* gene in addition to an increased expression of the *TLR4* gene (**Figure 4**).

#### 3.4.2 Immune signalling pathways

The transcripts coding for interleukin-17-like protein (*IL-17*) and protein toll (*TOLL*) had elevated expression levels in all three bacterial treatment groups (“Vcor Only”, “18 hr PB Only”, “18 hr PB + Vcor”) compared to the “Larvae Only” group (**Supplementary Table 5**). Genes responsible for producing other immune signalling molecules showed higher expression levels in all three treatment groups, including tumour necrosis factor receptor superfamily member 27 (*TNFR27*) and Cis-aconitate decarboxylase (*CAD*), indicating the activation and regulation of *NF-kB* and *MAPK* pathways (**Figure 4**).

In larvae treated with the probiotic combination (18 hr PB Only), transcripts for the myeloid differentiation primary response protein (*MyD88*) were found with increased expression levels compared to the “Larvae Only” control. Despite the increased expression of positive regulators of the *NF-kB* signalling pathway, the tripartite motif-containing protein 45-like (*TRIM45*) gene, a repressor of the *NF-kB* pathway, showed higher expression levels in the “18 hr PB Only” treatment group relative to the “Larvae Only” group (**Supplementary Table 5**). Alternatively, the E3 ubiquitin-protein ligase TRIM56-like (*TRIM56*) gene showed decreased expression in the probiotic-treated larvae (18 hr PB Only) (**Figure 4**). Additionally, the genes encoding the double-stranded RNA-specific adenosine deaminase (*ADAR*) protein and the complement C1q-like protein 4 (*C1q4*), involved in the cytosolic DNA-sensing and complement pathways, respectively, were found with higher expression in probiotic-treated larvae (18 hr PB Only) compared to the control group (Larvae Only) (**Figure 4**).

Larvae exposed to *V. coralliilyticus* (Vcor Only) had increased expression of the toll-like signalling regulator, tumour necrosis factor alpha-induced protein 3-like protein (*TNFAIP3*) relative to the “Larvae Only” control (**Figure 4**). Furthermore, other genes involved in signal regulating or contributing to signal transduction were differentially expressed in the “Vcor Only” control. For example, the 26S proteasome non-ATPase regulatory subunit 10 (*PSMD10*) and the serine/threonine-protein kinase RIO3 (*RIOK3*) both had an increased expression in *V. coralliilyticus*-infected larvae, compared to the “Larvae Only” control (**Figure 4**).

Larvae receiving both probiotic and pathogenic bacteria (18 hr PB + Vcor) had similar immune signalling gene expression profiles to the “18 hr PB Only” treatment group. For example, the *TRIM56* gene exhibited decreased expression while the genes encoding *MyD88*, *TRIM45*, *ADAR*, and *C1q4* all showed differentially increased expression in the “18 hr PB + Vcor” treatment group compared to the “Larvae Only” control (**Figure 4**).

#### 3.4.3 Immune effectors

Bacterial exposure influenced the gene expression of multiple immune effectors in all three treatment groups. For example, transcripts for the cell-surface mucin, integumentary mucin C.1 protein (*Muc.C1*) were elevated in expression in all treatment groups (“Vcor Only”, “18 hr PB Only”, and “18 hr PB + Vcor”) when compared to the “Larvae Only” control (**Figure 4**; **Supplementary Table 5**). Similarly, several transcripts coding for the multiple epidermal growth factor-like domains protein 10 (*MEGF10*) and the dual oxidase 2 protein (*DUOX2*) had elevated expression in all treatment groups (**Supplementary Table 4**).

In contrast, larvae treated with probiotics alone (18 hr PB Only) exhibited differential expressions of transcripts coding for the mucin 2 (*Muc2*) and mucin 5AC (*Muc5AC*) proteins. Additionally, the serine protease inhibitor Cvsi-2 gene (*Cvsi-2*) showed an elevated expression in the probiotic-treated larvae compared to the “Larvae Only” control group (**Supplementary Table 4**).

The “Vcor Only” treatment group resulted in the increased expression of the fos-related antigen-1 (*FOSL1*) gene when compared to the “Larvae Only” control. In contrast, the “18 hr PB + Vcor” treatment group did not result in differential expression of the *FOSL1* gene (**Supplementary Table 4**).

#### 3.4.4 Other immune/inflammatory genes

Larvae receiving bacterial additions (“Vcor Only”, “18hr PB Only”, and “18 hr PB + Vcor”) resulted in the increased expression of the cell-death inducing p53 (*CDIP53*) and the neuroglian genes when compared to the “Larvae Only” control (**Supplementary Table 5**). However, other genes involved in the regulation of apoptosis, including cell-death abnormality protein 1 (*CDAP1*) and the GTPase IMAP family members 4 and 7-like proteins (*GIMAP4*, *GIMAP 7*), had a decreased gene expression in the “18hr PB Only” treatment group when compared to the “Larvae Only” control (**Supplementary Table 4**). Additionally, the malignant brain tumours 1 protein gene (*DMBT1*) had an increased expression in the “18hr PB Only” treatment group compared to the “Larvae Only” control (**Figure 4**).

The differential expression of several inflammatory proteins was found to be unique to the “Vcor Only” treatment group. This included increased gene expression of the baculoviral IAP repeat-containing proteins 2, 3, and 7 (*BIRC2*, *BIRC3*, and *BIRC7*), the 2’,5’-phosphodiesterase 12 (*PDE12*), and the CCAAT/enhancer-binding protein beta (*CEBPB*) in pathogen-challenged larvae compared to the “Larvae Only” negative control (**Figure 4**).

Larvae exposed to both the probiotic and pathogenic bacteria (18 hr PB + Vcor) exhibited gene expressions comparable to that of the “18 hr PB Only” treatment group. For example, the *CDAP1*, *GIMAP4*, and *GIMAP7* genes showed decreased expression levels, while the *DMBT1* gene showed increased expression levels compared to the “Larvae Only” control (**Figure 4**).

## 4 Discussion

This study describes gene expression changes and survival of three- and four-day-old *C. gigas* larvae after exposure to probiotic and/or pathogenic bacteria. Larvae infected with *V. coralliilyticus* survive better when pre-treated with the probiotic combination (22). The current study supports this even when the pre-treated larvae were rinsed and transferred into probiotic-free autoclaved seawater. Hence, these results suggest a mechanism of action aside from direct inhibition of the pathogen by competitive exclusion. However, the results from the RNA sequencing were produced from larvae that were not rinsed and returned to probiotic-free seawater before infection. Therefore, inhibition and exclusion of the pathogen via direct interaction with the probiotics should not be discounted from interpretation.

Our data are compatible with mechanisms whereby probiotics may mitigate the effects of pathogenic infection of the larvae through immune stimulation, enhanced cellular barrier function, and reduced inflammation. We hypothesize that immune priming may be the primary mechanism responsible for the beneficial effects of the probiotic treatment observed here. Our treatments resulted in differential gene expression similar to those found in other studies that have exposed adult oysters to poly(I:C), which mimics viral double-stranded DNA to study immune priming (33, 34). Genes with similar expression patterns include the double-stranded RNA-specific adenosine deaminase (*ADAR*), tripartite motif-containing proteins (*TRIM45*, *TRIM56*), toll-like receptors (*TLR*s), tumour necrosis factor receptor (*TNFR27*), and various lectins. Overall, our results suggest that the specific probiotic combination treatment used here induces pathogen-defence mechanisms in otherwise vulnerable oyster larvae.

### 4.1 Early exposure to probiotic bacteria increases survival of *C. gigas* larvae subsequently challenged by *V. coralliilyticus*

The addition of ASW1 to a previously identified probiotic combination treatment of three bacterial isolates (22) further improved the relative percent survival of *V. coralliilyticus*-challenged *C. gigas* larvae compared to an infection control with no probiotics. While the relative percent survival between the three-strain and the four-strain combination was not statistically different (**Supplementary Table 3**), the latter increased the survival of infected larvae after an exposure of only six hours.

The probiotic treatment may directly or indirectly affect immune priming, with the latter possibly resulting from the probiotics influencing the early development of the oyster gut microbiome. It has previously been observed that *C. gigas* larvae begin ingesting small particles around 22 hpf (unpublished data), which allows the larvae two hours to ingest the probiotic bacteria before the larvae are rinsed with autoclaved seawater.

Ultimately, more work is needed to better understand the effects of probiotic additions on the possible development of the oyster gut microbiome and to determine the role of these probiotic bacteria in stimulating immune processes. Regardless, this work highlights a potentially beneficial and practical tool for promoting the health of larvae in oyster hatcheries and provides evidence for potential explanatory mechanisms that can be experimentally tested.

### 4.2 Characterization of the larval defence response to probiotic and pathogenic bacteria

The current study’s results reveal that there is a generalized response to all exogenous additions of bacteria, regardless of whether they involved probiotic or pathogenic microbes. In addition to this generalized response to Gram-negative bacteria, we observed specific gene expression responses to each different bacterial treatment (i.e., “Vcor only”, “PB only”, and “PB + Vcor”).

**Figure 5:**
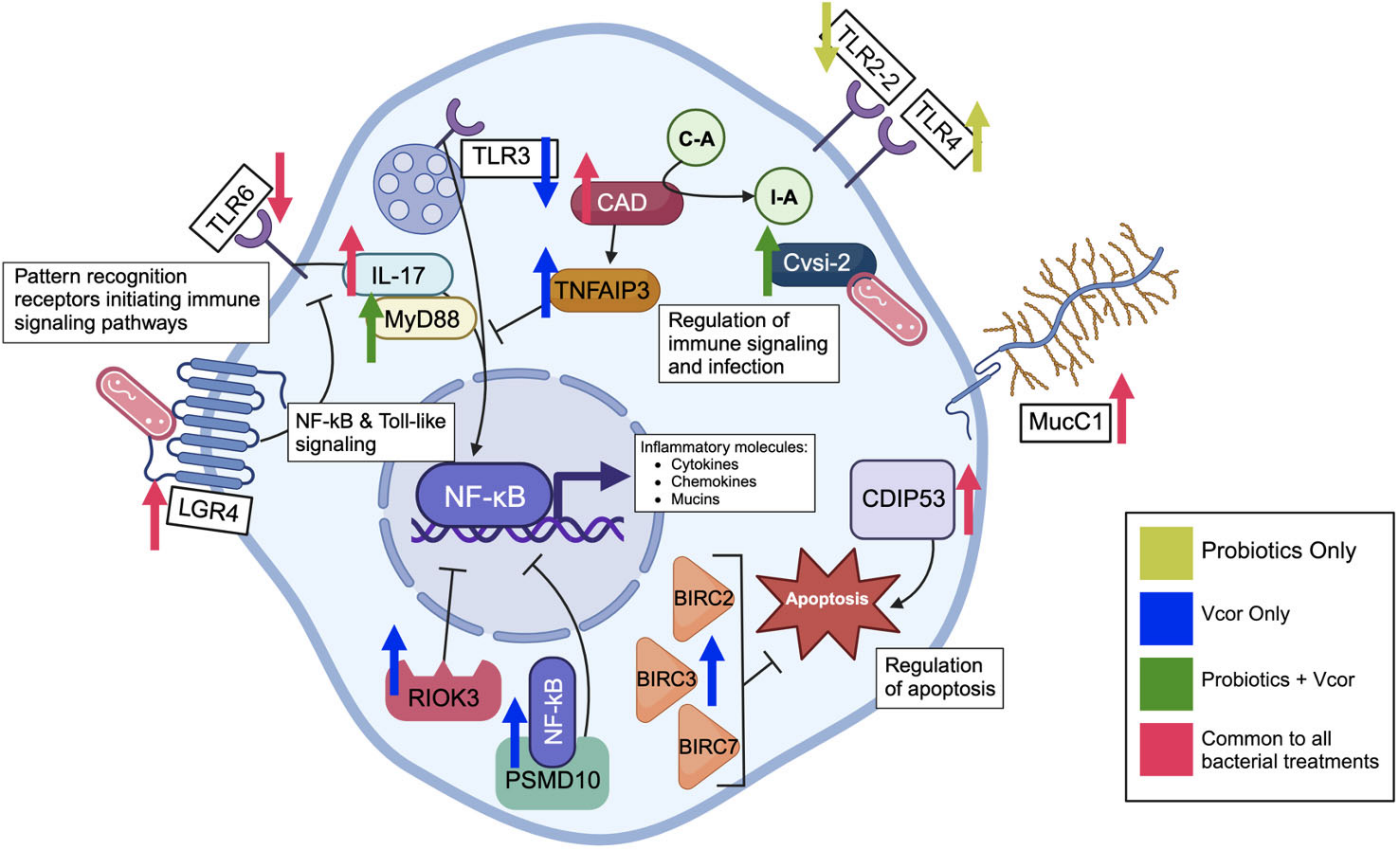
Defence-related genes in various cellular locations are differentially expressed in response to infection by *Vibrio coralliilyticus* and pre-treatment of the probiotic combination at 18 hours post-fertilization in *C. gigas* larvae. The arrows represent either an increase or decrease in gene expression caused by the exposure to the probiotic combination alone, *V. coralliilyticus* alone, *V. coralliilyticus* in addition to the probiotic treatment, or all three bacterial treatment groups. Pattern recognition receptors bind to the bacteria, sending an inflammatory signal through the *NF-kB* and toll-like signalling cascades. The activated *NF-kB* transcription factor leads to the production of cytokines, antimicrobial peptides, and immune effectors. Meanwhile, the tumour necrosis factor alpha-induced protein 3-like prevents activation of *NF-kB* transcription by inhibiting upstream signalling. The 26S proteasome non-ATPase regulatory subunit 10 binds to the *NF-kB* transcription factor and retains it in the cytosol as a negative regulator of the *NF-kB* signalling pathway. Additionally, the serine/threonine-protein kinase RIO3 inhibits *NF-kB* transcription, regulating inflammatory signalling. The immune effector serine protease inhibitor Cvsi2 directly interacts with endocytosed bacteria. Cell-surface mucins are produced and used as an inflammatory barrier of the cell. Apoptosis is enabled by the cell death-inducing p53-target protein 1 but inhibited by the baculoviral IAP repeat-containing proteins 2, 3, and 7.

It is generally understood that the first step of the bivalve innate immune response to foreign materials is for pattern recognition receptors to recognize and bind bacterial cell wall components, including lipopolysaccharides and peptidoglycans (12). Accordingly, the expression of multiple pattern recognition receptors involved in toll-like signalling was influenced by bacterial exposure in the following ways: 1) larvae exposed to any exogenous bacteria decreased expression of *TLR6*; 2) larvae that received probiotics at 18 hpf, regardless of whether they were exposed to *V. coralliilyticus* or not, demonstrated an additional decreased expression of *TLR2-2* and *TLR4*. The coordinated expression of these receptors is supported by previous reports that *TLR2* cooperates with *TLR6* (35) and heterodimerizes with *TLR4* in response to Gram-negative bacterial exposure (36); however, our results contradict previous studies, which reported upregulation of said toll-like receptors when *C. gigas* were exposed to live bacterial components, including peptidoglycan and lipopolysaccharides (37–39). Furthermore, larvae exposed to pathogenic *V. coralliilyticus* without any probiotic addition (Vcor Only) decreased expression of *TLR3*. In contrast to *TLR2-2*, *TLR4*, and *TLR6*, *TLR3* proteins are found intracellularly, localized within the endosome organelle where they recognize viral double-stranded DNA, signalling for it to be internalized and then transported to the lysosome (40). The reasons for decreased expressions of the *TLR* genes are unclear; however, possible explanations include the increased expression of genes that code for proteins responsible for the negative regulation of *TLR*s. For instance, the leucine-rich repeat-containing G-protein coupled receptor 4 (*LGR4*) protein is hypothesized to be a negative regulator of toll-like signalling and was found to have elevated expression in all three probiotic treatment groups (“18 hr PB Only”, “18 hr PB + Vcor”, and “Vcor Only”) (**Figure 5**). In addition, larvae infected with *V. coralliilyticus* that did not receive any probiotic addition (Vcor Only) experienced an upregulation of the toll-like signalling negative regulator, tumour necrosis factor alpha-induced protein 3-like (*TNFAIP3*), compared to the larvae that did not receive any bacterial additions (Larvae Only). The differential expression of this protein seems unique to the larval response to *V. coralliilyticus*, as it was not differentially expressed in response to the probiotics. In contrast, both probiotic and pathogen exposure increased the expression of *CAD*, another negative regulator of toll-like receptors. The *CAD* protein works as a negative regulator of toll-like receptors by stimulating the expression of *TNFAIP3* via *CAD*-dependent production of reactive oxygen species (ROS), leading to suppressed expression of toll-like receptors (**Figure 5**). Previously, itaconic acid, a by-product of cis-aconitate decarboxylase, has been found to inhibit ROS produced by phagocytes, therefore, regulating the innate immune response (41, 42). However, whether itaconic acid was increased in response to bacterial additions in this study is unknown. Van Nguyen & Alfaroo (43) found that adult mussels infected with a *V. coralliilyticus*/*neptunius*-like isolate experienced significantly higher ROS levels than non-infected individuals 6, 18, and 60 hours post-infection but that itaconic acid levels did not increase until 60 hours post-infection. Therefore, it is possible that increased expression of itaconic acid could occur in infected larvae, but not before 60 hours after exposure to *V. coralliilyticus* (108 hours post-fertilization).

Ultimately, even though multiple toll-like receptors had a lower expression level in the bacteria-treated larvae, downstream signalling molecules whose activation is typically induced via toll-like receptors, including interleukin-17 (*IL-17*), had significantly increased expression. *IL-17* transcripts are expressed in the gills of *Mytilus galloprovincialis* in response to infection by *Vibrio splendidus*, suggesting a contribution to mucosal immunity (44). Additionally, *IL-17* is known to be directly responsive to bacterial LPS and contributes to the activation of both myeloid differentiation primary response 88 (*MyD88*) and the *NF-kB* pathway (**Figure 5**). *MyD88*, found with elevated expression in larvae exposed to the probiotics, is directly stimulated by most toll-like receptors and plays a central role in the innate immune response, activating IL-1R associated kinases (*IRAK*) and subsequently the *NF-kB* and mitogen-activated protein kinase (*MAPK*) pathways (45). Therefore, *MyD88* might be stimulated by long-term bacterial exposure as opposed to toll-like receptors whose expression may decline with continuous bacterial exposure (46).

In the absence of probiotics, infection by *V. coralliilyticus* caused an increase in expression of the 26S proteasome non-ATPase regulatory subunit 10 protein (*PSMD10*) and serine/threonine-protein kinase RIO3 (*RIOK3*), both of which have been found to inhibit *NF-kB* activation (**Figure 5**). For example, *PSMD10* has been hypothesized to retain *NF-kB* in the cytoplasm of cells, subsequently inhibiting *NF-kB* activity, whereas *RIOK3* has been shown to inhibit TNF-alpha and caspase-10 induced activation of the *NF-kB* pathway (47, 48). However, when larvae were exposed to both the probiotics and *V. coralliilyticus*, neither the *RIOK3* nor *PSMD10* genes were found to be differentially expressed. Therefore, this result may be due to the probiotics directly inhibiting *V. coralliilyticus* growth, virulence, or both, consequently preventing the initial upregulation of these two genes. Alternatively, some of the numerous uncharacterized DEGs influenced by the probiotics may function as regulators of *RIOK3* and *PSMD10*. Further progress in the characterization of the *C. gigas* genome will be required to evaluate this possibility.

In addition to immune signalling pathways, treatment with both probiotic and pathogenic bacteria influenced the expression of various inflammation and effector molecules, including mucins. Elevated Mucin C1 (*Muc.C1*) expression was seen in all larvae exposed to bacteria; however, larvae treated with probiotics had particularly elevated levels of Mucin 2 (*Muc2*) and Mucin 5AC (*Muc5AC*) expression (**Figure 5**). These results correspond with previously identified localizations and proposed functions of each mucin. For example, *Muc.C1* has been recognized as a transmembrane protein in many cell types, including human immune cells (49). Furthermore, *Muc.C1* has been observed to behave as a binding site for bacterial pathogens, such as *Pseudomonas aeruginosa.* Following the binding of bacteria, *Muc.C1* contributes to an anti-inflammatory response characterized by the inhibition of toll-like signalling (50). The resulting decreased expression of toll-like receptors identified in this study agrees with *Muc.C1*’s role within the anti-inflammatory process. In contrast, *Muc2* and *Muc5AC* are mainly localized to the digestive tract and contribute to maintaining a mucosal barrier that will protect tissue surfaces and aid in removing unwanted material, including bacteria (49, 51, 52).

Aside from immune effectors, several proteins involved in apoptosis experienced differential expression due to bacterial exposure (**Figure 5**). For example, the cell-death-inducing p53 (*CDIP53*) protein had elevated expression in all bacteria-exposed larvae; this protein induces cellular apoptosis through the intrinsic pathway (53). In contrast, probiotic exposure seems to prevent apoptosis as there was a reduced expression of GTPase immune-associated proteins 4 and 7 (*GIMAP4*, *GIMAP7*) (apoptosis accelerators) and cell-death abnormality protein 1-like (a protein responsible for enabling phagocytes to engulf apoptotic cells) (54–57). Reduced apoptosis regulators suggest a reduced need for cell death as a defence mechanism. These results align with previous studies that evaluated the impacts of probiotic bacteria on bivalve larvae, including the Eastern oyster, *Crassostrea virginica* (4). Additionally, larvae infected with *V. coralliilyticus* displayed increased expression of various baculoviral IAP repeat-containing proteins (*BIRC*s), which are negative regulators of apoptosis. *BIRC*s present anti-apoptotic features by mediating multiple caspases, including caspase-3, 7, and 9 (58). The lack of self-induced apoptosis by *V. coralliilyticus*-infected cells might allow the pathogen to replicate and overwhelm this defence mechanism.

## 5 Conclusion

The current study describes the immune response to infection of *C. gigas* larvae by *V. coralliilyticus* and the modulation of this response by the application of a combination of probiotics that improved the survival of the Vibrio-challenged larvae. When larvae were pre-treated with the probiotics at a total concentration of 3.0 x 10^5^ CFU/mL, gene expression patterns related to *V. coralliilyticus* exposure were suppressed, and the probiotic treatment stimulated inflammatory molecules supportive of an immune response that likely reduced the detrimental effects of *V. coralliilyticus* infection on larval survival. Further research should focus on the mechanics of beneficial bacteria that protect *C. gigas* larvae and other cultured bivalve species against microbial pathogens and improve the effectiveness of these and similar probiotic treatments in bivalve hatcheries.

## Data availability statement

The original contributions presented in the study are included in the article/**Supplementary Materials**. Further inquiries can be directed to the corresponding author.

## Ethics statement

The manuscript presents research on animals that do not require ethical approval for their study.

## Author contributions

JH: Data curation, Formal analysis, Investigation, Methodology, Visualization, Writing – original draft, Writing – review & editing. RM: Conceptualization, Methodology, Supervision, Writing – review & editing, Funding acquisition. CL: Conceptualization, Funding acquisition, Methodology, Resources, Supervision, Writing – review & editing. CS: Conceptualization, Funding acquisition, Resources, Supervision, Writing – review & editing, Project administration.
