# Corrigendum: Immunomodulatory effects of a probiotic combination treatment to improve the survival of Pacific oyster (*Crassostrea gigas)* larvae against infection by *Vibrio coralliilyticus*

**DOI:** 10.3389/fimmu.2024.1454221

**Published:** 2024-07-09

**Authors:** Jennifer Hesser, Ryan Mueller, Chris Langdon, Carla Schubiger

**Affiliations:** ^1^ Department of Biomedical Sciences, Carlson College of Veterinary Medicine, Oregon State University, Corvallis, OR, United States; ^2^ Department of Microbiology, College of Science, Oregon State University, Corvallis, OR, United States; ^3^ Coastal Oregon Marine Experiment Station and Department of Fisheries, Wildlife, and Conservation Sciences, College of Agricultural Sciences, Oregon State University, Corvallis, OR, United States

**Keywords:** Pacific oyster larvae, probiotics, immune response, *Vibrio coralliilyticus*, aquaculture

In the published article, there was an error. Under **Author contributions** section, “conceptualization” was not included for author CS.

A correction has been made to **Author contributions.** This sentence previously stated:

“CS: Funding acquisition, Resources, Supervision, Writing – review & editing, Project administration.”

The corrected sentence appears below:

“CS: Conceptualization, Funding acquisition, Resources, Supervision, Writing – review & editing, Project administration.”

The authors apologize for this error and state that this does not change the scientific conclusions of the article in any way. The original article has been updated.

