# Supplementary material for: Immunomodulatory effects of a probiotic combination treatment to improve the survival of Pacific oyster (Crassostrea gigas) larvae against infection by Vibrio coralliilyticus
Source: Front Immunol. 2024 Apr 8;15:1380089. doi: 10.3389/fimmu.2024.1380089 (PMC11033467; doi:10.3389/fimmu.2024.1380089)
Supplement: Supplementary file 1 [file DataSheet_1.docx]

**References for Supplementary Table S_5**

1. Gerdol M, Venier P, Pallavicini A. The genome of the pacific oyster Crassostrea gigas brings new insights on the massive expansion of the C1q gene family in Bivalvia. Dev Comp Immunol. (2015) 49(1):59–71. doi: 10.1016/j.dci.2014.11.007

2. Du B, Luo W, Li R, Tan B, Han H, Lu X, et al. Lgr4/Gpr48 negatively regulates TLR2/4-associated pattern recognition and innate immunity by targeting CD14 expression. J Biol Chem. (2013) 288(21):15131–41. doi: 10.1074/jbc.M113.455535

3. Brightbill HD, Libraty DH, Krutzik SR, Yang RB, Belisle JT, Bleharski JR, et al. Host defense mechanisms triggered by microbial lipoproteins through toll-like receptors. Science. (1999) 285(5428):732–6. doi: 10.1126/science.285.5428.732

4. Jin MS, Kim SE, Heo JY, Lee ME, Kim HM, Paik SG, et al. Crystal Ssructure of the TLR1-TLR2 heterodimer induced by binding of a tri-acylated lipopeptide. Cell. (2007) 130(6):1071–82. doi: 10.1016/j.cell.2007.09.008

5. Kawasaki T, Kawai T. Toll-like receptor signaling pathways. Front Immunol. (2014) 5:461. doi: 10.3389/fimmu.2014.00461

6. Sameer AS, Nissar S. Toll-like receptors (TLRs): structure, functions, signaling, and role of their polymorphisms in colorectal cancer susceptibility. BioMed Res Int. (2021) 2021:1157023. doi: 10.1155/2021/1157023

7. Li Y, Zhang P, Wang C, Han C, Meng J, Liu X, et al. Immune responsive gene 1 (IRG1) promotes endotoxin tolerance by increasing A20 expression in macrophages through reactive oxygen species. J Biol Chem. (2013) 288(23):16225–34. doi: 10.1074/jbc.M113.454538

8. de Reuver R, Maelfait J. Novel insights into double-stranded RNA-mediated immunopathology. Nat Rev Immunol. (2023) 1–15. doi: 10.1038/s41577-023-00940-3

9. Yu J, Teng S, Yue X, Wang H, Liu B. The Toll pathway and Duox-ROS system are required for the clam antibacterial immune response in the hepatopancreas. Aquaculture. (2023) 574:739637. doi: 10.1016/j.aquaculture.2023.739637

10. Fu L, Zhou X, Jiao Q, Chen X. The functions of TRIM56 in antiviral innate immunity and tumorigenesis. Int J Mol Sci. (2023) 24(5):5046. doi: 10.3390/ijms24055046

11. Rosani U, Varotto L, Gerdol M, Pallavicini A, Venier P. IL-17 signaling components in bivalves: comparative sequence analysis and involvement in the immune responses. Dev Comp Immunol. (2015) 52(2):255–68. doi: 10.1016/j.dci.2015.05.001

12. Janssens S, Beyaert R. A universal role for MyD88 in TLR/IL-1R-mediated signaling. Trends Biochem Sci. (2002) 27(9):474–82. doi: 10.1016/S0968-0004(02)02145-X

13. Shan J, Wang P, Zhou J, Wu D, Shi H, Huo K. RIOK3 interacts with caspase-10 and negatively regulates the NF-κB signaling pathway. Mol Cell Biochem. (2009) 332(1–2):113–20. doi: 10.1007/s11010-009-0180-8

14. NCBI. LOC105334196 protein toll [Crassostrea gigas (Pacific oyster)](2024). https://www.ncbi.nlm.nih.gov/gene/?term=LOC105334196

15. Jin Z, Zhu Z. The role of TRIM proteins in PRR signaling pathways and immune-related diseases. Int Immunopharmacol. (2021) 98:107813. doi: 10.1016/j.intimp.2021.107813

16. Shibata M, Sato T, Nukiwa R, Ariga T, Hatakeyama S. TRIM45 negatively regulates NF-κB-mediated transcription and suppresses cell proliferation. Biochem Biophys Res Commun. (2012) 423(1):104–9. doi: 10.1016/j.bbrc.2012.05.090

17. Wullaert A, Verstrepen L, Huffel SV, Adib-Conquy M, Cornelis S, Kreike M, et al. LIND/ABIN-3 is a novel lipopolysaccharide-inducible inhibitor of NF-κB activation. J Biol Chem. (2007) 282(1):81–90. doi: 10.1074/jbc.M607481200

18. Sinha SK, Zachariah S, Quiñones HI, Shindo M, Chaudhary PM. Role of TRAF3 and -6 in the activation of the NF-kappa B and JNK pathways by X-linked ectodermal dysplasia receptor. J Biol Chem. (2002) 277(47):44953–61. doi: 10.1074/jbc.M207923200

19. Chen Y, Li HH, Fu J, Wang XF, Ren YB, Dong LW, et al. Oncoprotein p28 GANK binds to RelA and retains NF-kappaB in the cytoplasm through nuclear export. Cell Res. (2007) 17(12):1020–9. doi: 10.1038/cr.2007.99

20. Kinoshita S, Akira S, Kishimoto T. A member of the C/EBP family, NF-IL6 beta, forms a heterodimer and transcriptionally synergizes with NF-IL6. Proc Natl Acad Sci. (1992) 89(4):1473–6. doi: 10.1073/pnas.89.4.147

21. Roy SK, Hu J, Meng Q, Xia Y, Shapiro PS, Reddy SPM, et al. MEKK1 plays a critical role in activating the transcription factor C/EBP-β-dependent gene expression in response to IFN-γ. Proc Natl Acad Sci. (2002) 99(12):7945–50. doi: 10.1073/pnas.12207579

22. End C, Bikker F, Renner M, Bergmann G, Lyer S, Blaich S, et al. DMBT1 functions as pattern-recognition molecule for poly-sulfated and poly-phosphorylated ligands. Eur J Immunol. (2009) 39(3):833–42. doi: 10.1002/eji.200838689

23. Mollenhauer J, Herbertz S, Helmke B, Kollender G, Krebs I, Madsen J, et al. Deleted in malignant brain tumors 1 is a versatile mucin-like molecule likely to play a differential role in digestive tract cancer. Cancer Res. (2001) 61(24):8880–6.

24. Wang L, Sun J, Wu Z, Lian X, Han S, Huang S, et al. AP-1 regulates the expression of IL17-4 and IL17-5 in the pacific oyster Crassostrea gigas. Fish Shellfish Immunol. (2020) 97:554–63. doi: 10.1016/j.fsi.2019.12.080

25. Dhar P, McAuley J. The role of the cell surface mucin MUC1 as a barrier to infection and regulator of inflammation. Front Cell Infect Microbiol. (2019) 9:452801. doi: 10.3389/fcimb.2019.00117

26. Williams MJ. The Drosophila cell adhesion molecule Neuroglian regulates Lissencephaly-1 localisation in circulating immunosurveillance cells. BMC Immunol. (2009) 10:17. doi: 10.1186/1471-2172-10-17

27. Cartier J, Berthelet J, Marivin A, Gemble S, Edmond V, Plenchette S, et al. Cellular inhibitor of apoptosis protein-1 (cIAP1) can regulate E2F1 transcription factor-mediated control of cyclin transcription. J Biol Chem. (2011) 286(30):26406–17. doi: 10.1074/jbc.M110.191239

28. Zhou AY, Shen RR, Kim E, Lock YJ, Xu M, Chen ZJ, et al. IKKε-mediated tumorigenesis requires K63-linked polyubiquitination by a cIAP1/cIAP2/TRAF2 E3 ubiquitin ligase complex. Cell Rep. (2013) 3(3):724–33. doi: 10.1016/j.celrep.2013.01.031

29. Bertrand MJM, Lippens S, Staes A, Gilbert B, Roelandt R, De Medts J, et al. cIAP1/2 are direct E3 ligases conjugating diverse types of ubiquitin chains to receptor interacting proteins kinases 1 to 4 (RIP1-4). PloS One. (2011) 6(9):e22356. doi: 10.1371/journal.pone.0022356

30. Kasof GM, Gomes BC. Livin, a novel inhibitor of apoptosis protein family member. J Biol Chem. (2001) 276(5):3238–46. doi: 10.1074/jbc.M003670200

31. Ma L, Huang Y, Song Z, Feng S, Tian X, Du W, et al. Livin promotes Smac/DIABLO degradation by ubiquitin–proteasome pathway. Cell Death Differ. (2006) 13(12):2079–88. doi: 10.1038/sj.cdd.4401959

32. Brown L, Ongusaha PP, Kim H, Nuti S, Mandinova A, Lee JW, et al. CDIP, a novel pro‐apoptotic gene, regulates TNFα‐mediated apoptosis in a p53‐dependent manner. EMBO J. (2007) 26(14):3410–22. doi: 10.1038/sj.emboj.7601779

33. Limoges MA, Cloutier M, Nandi M, Ilangumaran S, Ramanathan S. The GIMAP family proteins: an incomplete puzzle. Front Immunol. (2021) 12: 679739. doi: 10.3389/fimmu.2021.679739

34. Kubota K, Nakahara K, Ohtsuka T, Yoshida S, Kawaguchi J, Fujita Y, et al. Identification of 2’-phosphodiesterase, which plays a role in the 2-5A system regulated by interferon. J Biol Chem. (2004) 279(36):37832–41. doi: 10.1074/jbc.M400089200
